# A Scoping Review of Communicating Neuropsychological Test Results to Patients and Family Members

**DOI:** 10.1007/s11065-021-09507-2

**Published:** 2021-04-20

**Authors:** Angélique AA Gruters, Inez HGB Ramakers, Frans RJ Verhey, Roy PC Kessels, Marjolein E de Vugt

**Affiliations:** 1 Department of Psychiatry and Neuropsychology, School for Mental Health and Neuroscience, Alzheimer Center Limburg, Maastricht University, Maastricht, the Netherlands; 2 Donders Institute for Brain, Cognition and Behaviour, Radboud University, Nijmegen, the Netherlands; 3 Department of Medical Psychology & Radboudumc Alzheimer Center, Radboud University Medical Center, Nijmegen, the Netherlands

**Keywords:** *Neuropsychological feedback*, *Neuropsychological assessment*, *Communication*, *Neuropsychology*, *Patient outcome*

## Abstract

Feedback of neuropsychological test results to patients and family members include psychoeducation and implications for daily life. This scoping review aimed to provide an overview of the literature on neuropsychological feedback and to offer clinical recommendations. In accordance with formal scoping review methodology, PubMed, PsycInfo, Web of Science, CINAHL, and Embase databases were searched. Studies were included if they reported on neuropsychological feedback, if full papers were available, and if they included human participants. All languages were included, and no limit was placed on the year of publication. Of the 2,173 records screened, 34 publications met the inclusion criteria. Five additional publications were included after cross-referencing. An update of the search led to the inclusion of two additional papers. Of these 41 publications, 26 were research papers. Neuropsychological feedback is provided for a wide spectrum of diagnoses and usually given in-person and has been related to optimal a positive effect on patient outcomes (e.g. increase the quality of life). Most papers reported on satisfaction and found that satisfaction with an NPA increased when useful feedback was provided. However, information retention was found to be low, but communication aids, such as written information, were found to be helpful in improving retention. The current review demonstrated the benefits of neuropsychological feedback and that this should be part of standard clinical procedures when conducting a neuropsychological assessment. Further research on the benefits of neuropsychological feedback and how to improve information provision would enrich the neuropsychological literature.

## Background

An important role of healthcare providers is to deliver feedback from diagnostic findings and medical information to patients. A neuropsychological assessment (NPA) evaluates the cognitive performance of a patient and can provide insight into cognitive strengths and weaknesses (Lezak et al., 2012). In this study, explaining neuropsychological assessment results to patients and family members is defined as neuropsychological feedback. To our knowledge, this definition has been predominantly used in the literature. However, other terms have also been used (e.g., neuropsychological testing feedback). The goal of neuropsychological feedback is to help patients and family members understand the results and the implications for daily life functioning (Postal & Armstrong, 2013). Furthermore, neuropsychological feedback gives the opportunity to evaluate rehabilitation or treatment planning, provide support to patients and family members who might experience difficulties with adapting to a diagnosis, offer guidelines for decision-making, and answer questions or concerns patients may have about their NPA results (Brenner, 2003; Gorske & Smith, 2009). Giving feedback has been recommended by international clinical research groups and ethical guidelines state that a psychologist must undertake a reasonable attempt to explain the results of their assessment (Baxendale et al., 2019; Wilson et al., 2015; American Psychological Association, 2017; Baxendale & Thompson, 2010). Traditionally, neuropsychological feedback received little scientific attention and was not always part of clinical practice. Only a few studies at the end of the 80s have described the possible added value of giving neuropsychological feedback, and argued that patients who received valuable feedback were more satisfied with the NPA (Allen et al., 1986; Bennett-Levy et al., 1994). Other authors evaluated the effect of receiving personalized information in a group of 28 patients, which also included neuropsychological test results, and demonstrated that this resulted in to greater effort in therapy and more satisfaction with rehabilitation treatment (Pegg et al., 2005). Nearing the end of the 00s the number of publications increased that focused solely on the benefits of neuropsychological feedback. It was also shown that neuropsychological feedback has since become more embedded in standard practice (Westervelt et al., 2007). In a recent study in 218 patients from a neuropsychological outpatient clinic, neuropsychological feedback was shown to lead to improved quality of life, better comprehension of the medical condition, and an improved ability to cope with that condition (Rosado et al., 2018). In another study in 31 patients with ADHD or a mood disorder, providing feedback also resulted in less psychiatric and cognitive symptoms, and improved self-efficacy (Lanca et al., 2019). To our knowledge, research evaluating the benefits of neuropsychological feedback is limited. Due to the increase in publications regarding this topic in the past ten years a scoping review is warranted. It is important to gain more insight into what is known about neuropsychological feedback to improve quality of care. This scoping review aims to provide an overview (e.g., study types, methods used, results, quality of papers) about neuropsychological feedback. Furthermore, another aim is to offer recommendations for clinical practice.

## Methods

### Design

A preliminary literature search resulted in diversity of methods and multiple sources concerning neuropsychological feedback. Consequently, a scoping review was chosen over a systematic review due to the broader approach, as scoping reviews include multiple sources, such as studies with different designs, opinion or position papers, and gray literature (Arksey & O'Malley, 2005; Peters et al., 2015). A scoping review is used to provide an overview of the literature in the area of interest, to identify gaps of knowledge in the evidence base and to summarize relevant findings (Arksey & O'Malley, 2005). The current review was guided by the methodological framework described by Arksey and O'Malley (2005) and additional recommendations of Levac et al. (2010). This framework consisted of five stages guiding the scoping process of identifying the research question, identifying relevant studies, study selection, charting the data, and summarizing and reporting the results (Levac et al., 2010). Furthermore, the PRISMA checklist for scoping reviews was used as reporting guideline (Tricco et al., 2018). Although a quality appraisal is not often applied in scoping reviews, we opted to use the Mixed Method Appraisal Tool 2018 version (Pace et al., 2012). This is a reliable and efficient critical appraisal tool with five criteria per research design to review the quality of methodology in systematic reviews and has been used in prior scoping reviews (Breneol et al., 2017; Bieber et al., 2019).

### Inclusion and Exclusion Criteria

We included books, book chapters, and research articles reporting on providing neuropsychological feedback to patients or family members (e.g., not studies that focused on broader assessment practices such as rehabilitation programs). All types of research designs, patient groups, and languages were included. No restrictions were made on year of publication. Research papers were excluded when no results were reported on neuropsychological feedback. Books, book chapters and opinion or position papers were excluded if neuropsychological feedback was not included as the main topic. Papers were also excluded if no full paper was available (e.g., conference abstracts) or in the case of nonhuman studies.

### Data Sources and Search Strategy

We searched the following databases: PubMed, PsycInfo, Web of Science, CINAHL, and Embase. A combination of free text terms in Title/Abstract and descriptor terms (e.g., MeSH terms) were used in the search string. The full search strategy for each database is provided in the supplementary material. The literature search was carried out on December 9, 2019. The search was updated on June 11, 2020.

### Study Selection

Two authors (AG, IR) independently screened the titles and abstracts. They met in person after having screened the first 50 abstracts to discuss challenges and uncertainties related to study selection. After completion of abstract screening, the interrater reliability was therefore excellent (Cohen’s *k* = 0.89) (Altman, 1990). Afterwards, one author (AG) evaluated all full texts independently to determine eligibility for inclusion. The second author (IR) screened 10% (*n* = 8) of all full texts, randomly selected. This subsample was independently evaluated and no rater overlap was present. The interrater reliability was also excellent (Cohen’s *k* = 1.00). When it was uncertain whether to include a full text, this was discussed with the second author. Cross-referencing was used to determine if other relevant publications should be included.

### Charting of Data

One of the reviewing authors (AG) extracted and summarized the data from the included publications. A data extraction plan was piloted for applicability and completeness and discussed among the other authors. The following was included in the chart: study design, setting, study population, sample size, methodology, intervention type and comparator, outcomes, outcome measurements, analysis, characteristics of NPA, characteristics of neuropsychological feedback, framework for feedback, key findings related to neuropsychological feedback, and key findings related to aids in neuropsychological feedback. The main topics were analyzed (qualitative content approach) and thematically classified and narratively described. The first draft of topics was discussed among the authors until consensus was reached. Quantitative analyses were not conducted due to the diversity of studies and the descriptive nature of most studies.

## Results

**Fig. 1:**
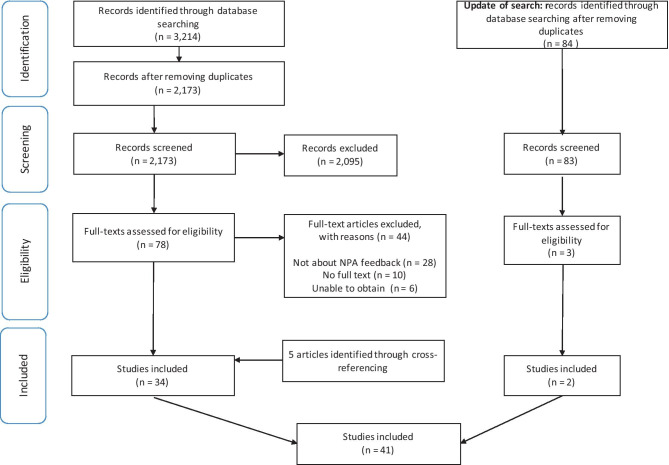
Flow diagram of the current scoping review

The search yielded a total of 3,214 records; 2,173 remained after duplicates were removed. After screening the abstracts, 78 papers were evaluated for full text screening. A total of 34 papers were included, and five additional papers were identified through cross-referencing. The search was updated on June 11, 2020, which led to 84 extra records that were screened and three that underwent full text screening. Two papers were included from the updated search. Figure 1 shows the flowchart of the selection process. In this study, the findings from 41 papers are summarized using a narrative report. An overview of the characteristics and outcomes of these publications is described in Table 1.

**Table 1:** Summary of included publications

Reference Type of document	Purpose/aim	Design (D) Population (P) Setting (S)	Methods (M) Intervention type (I) Relevant outcomes (O)	Relevant results/recommendations
Allen et al., 1986 Position paper	Advocate a collaborative process approach to NPA and provide guidelines for discussing the results with psychiatric patients and family members	P: Psychiatric patients and family members	N/A	Advice to begin with in-person feedback to evaluate misunderstandings and emotional reactions.
Arffa & Knapp, 2008 Research paper	Examine utility and value ratings of NPA by measuring parent perceptions, as well as determining whether testing led to diagnostic changes or recommendations	D: quantitative, descriptive P: 64 parents of children with complex neuro-developmental and acquired neurological disorders S: neurodevelopmental outpatient clinic	M: survey (70% response rate), medical records O: satisfaction with NPA, number of recommendations	Feedback was given in-person, 2 weeks to 2 months later. All received a written report. Greatest utility rating of NPA was understanding strengths and weaknesses. An average of 6.5 primary and 11.1 secondary recommendations were given.
Belciug, 2006 Research paper	Evaluate caregiver concerns after neuropsychological feedback	D: quantitative, descriptive P: 45 caregivers of patients with stroke	M: survey O: concern regarding patient’s level of functioning after NPA with feedback	Concerns related to safety, the future, dealing with emotional aspects, and what to do when the patient cannot perform a task independently were the most prominent after the feedback session.
Bennett-Levy et al., 1994 Research paper	To explore the consumer experience with an NPA and how neuropsychologists can improve their quality of service	D: quantitative, descriptive P: 129 patients with various diagnoses (most common head injury and stroke) S: 5 outpatient clinics (2 hospitals, 3 rehabilitation centers)	M: survey (51% response rate) O: experience with NPA	One influence on NPA consumer experience was whether they received feedback and if this was seen as useful. A total of 32% patients did not receive feedback, and when it was given, this was not always remembered (30%) or understood (24%). The majority found the feedback session useful (67%). Only 26% received feedback on paper.
Bodin et al., 2007 Research paper	Enhance understanding of parent satisfaction with pediatric neuropsychological evaluation	D: quantitative, descriptive P: 117 parents of children with various diagnoses (most common ADHD and epilepsy) S: outpatient clinic (children’s hospital)	M: survey (35% response rate) O: satisfaction with NPA	All received a written report. Overall, parents were satisfied and 54% found the session helpful. A total of 68% felt that it helped them address their child’s problems. The majority agreed that feedback helped in understanding the child’s strengths and suggested ways of addressing problems.
Carone et al., 2010 Opinion paper	Present conceptual framework for providing feedback regarding invalid responding or effort with recommendations for how to handle complaints	P: any diagnostic group	N/A	In-person feedback model with three phases (Table 3)
Carone et al., 2013 Opinion paper	Review and update symptom validity feedback model and describe feedback approach with patients reporting with persisting symptoms after MTBI	P: MTBI patients	N/A	In-person feedback model with three phases with additional tips for patients with MTBI (Table 3)
Carone, 2017 Opinion paper	Present a feedback model for patients who are not reassured by feedback because they blame external factors	P: patients with high level of cognitive complaints, but normal test performance	N/A	In-person feedback model with three phases (Table 3)
Cheung et al., 2014 Research paper	Explored parent and teacher understanding of neuropsychology reports, implementation rates for recommendations and their perceived effectiveness. Barriers were also evaluated	D: qualitative P: 17 parents of childhood who had a brain tumor and 8 teachers S: children’s hospital	M: semi-structured interviews O: perceived effectiveness of report, difficulty of implementing recommendations	Feedback was given in-person, all received written report. Majority of parents found the reports clear and comprehensive. Recommendations were evaluated as effective and easy to implement. A need for a more practical translation to daily life, a glossary for terminology, and a follow-up consultation was expressed.
Clement et al., 2001 Opinion paper	Describe use of a neuropsychology telemedicine clinic in an army medical center.	P: patients with neurological disorders or brain injury S: army medical center	N/A	Experiences with feedback via telemedicine were positive in areas where these services otherwise would not be available.
Connery et al., 2016 Research paper	Examine impact of neuropsychological consultation when invalid performance has been identified in a pediatric population. Additionally, provide a conceptual feedback model	D: quantitative, non-randomized P: 70 parents of children with history of MTBI S: outpatient pediatric concussion program	M: survey I: comparing group with noncredible effort and feedback model (n = 9) to those with no validity concerns present and feedback care as usual (n = 61) O: post-concussive symptom reduction, parent satisfaction with NPA	In both the group with the feedback model (noncredible effort) and the care as usual group (credible effort) similarly high levels of satisfaction were found. In the noncredible group, a greater reduction of self-reported symptoms was found after feedback compared to children with a credible performance.
Crosson, 2000 Book chapter	Describe applications of NPA results and summarizes problems arising when giving neuropsychological feedback and principles to use against these problems	Not specified	N/A	Describes potential pitfalls and solutions for delivering in-person feedback (Table 3).
Evans et al., 2019 Position paper	Highlight current challenges and barriers in composing neuropsychological reports and communicating key findings to Spanish speaking caregivers of school-aged Latino children	P: Spanish speaking caregivers of school-aged Latino children	N/A	Recommendations for report writing, such as language translation and considering both cultural and linguistic differences.
Fallows & Hilsabeck , 2013 Research paper	To see whether supplementing oral feedback with written information leads to greater retention of information and improved adherence to recommendations	D: quantitative, randomized P: 66 veterans with cognitive or no cognitive disorders S: veterans administration in a neuropsychology clinic	M: structured interview after feedback and 1 month later by phone I: group with oral feedback (n = 36) and group with oral feedback + written information (n =30) O: retention of diagnostic information, adherence to treatment recommendations	Group with both in-person feedback and supplemental written letter freely recalled more recommendations one month after feedback compared to the no letter group. Overall recall of recommendations and diagnostic information (with exception of knowing they had cognitive problems) was low.
Farmer & Brazeal, 1998 Research paper	Evaluate reaction of parents to their child’s NPA, specifically related to their parent perceptions of their child and their ability to cope with their child’s disease.	D: quantitative, descriptive P: 55 parents of children with neurodevelopmental problems S: outpatient clinic (hospital)	M: survey (47% response rate) O: effect of NPA on parent perceptions of their child, coping with child’s disability, adequacy of NPA	25% of parents found the in-person feedback session most helpful of NPA. Majority evaluated the report as most useful. Feedback increased understanding of child’s strengths and weaknesses and helped make a difference for their child. Strongest predictor of NPA satisfaction were perceptions of professional’s concern, technical competence, and rating of recommendations.
Foran et al., 2016 Research paper	Develop and pilot a measure of patient satisfaction that encompasses themes, activities, settings and interactions specific to NPA process	D: mixed-methods P: 81 patients with various diagnosis (most common chronic illness, psychiatric diagnosis or traumatic injury) S: neuropsychology outpatient clinic (hospital)	M: systematic search, focus groups, pilot study O: satisfaction with NPA	Overall, high satisfaction with NPA (79%). Satisfaction with testing (85%) higher than pre-assessment (77%) and feedback (68%). A total of 44% patients received feedback from a neuropsychologist, 38% from a third party and 18% no feedback. Critique on feedback was related to difficulty of understanding the information and emotional impact of diagnosis.
Gass & Brown, 1992 Opinion paper	General framework for presenting feedback is described with an emphasis on techniques designed to maximize patient benefit. Special issues are discussed	P: patients with brain injury	N/A	In-person feedback model with six phases (Table 3)
Gorske,^2007^ Position paper	Present a humanistic model for providing neuropsychological feedback	Not specified	N/A	In-person feedback model with five phases (Table 3)
Gorske & Smith, 2009 Book	Describe a client-centered approach in using NPA feedback therapeutically.	Not specified	N/A	In-person feedback model with five phases (Table 3)
Green, 2000 Book chapter	Discusses the final stage of NPA, provision of feedback to the patient and others, and planning of treatment interventions and follow-up services.	Not specified	N/A	Recommendations given for in-person feedback, such as that it is sometimes more appropriate to receive feedback from another professional or to ensure that the information is understood and accepted.
Griffin & Christie, 2008 Opinion paper	Discusses current approaches in NPA of children	P: children S: hospital- or community-based	N/A	An audit showed that most families found the report difficult to understand or unhelpful. Recommendations given for in-person feedback, such as communicating in an understandable manner and sending a written report with creative/child-friendly language.
Gruters et al., 2020 Research paper	Gain insight into experiences of patients and family members with an NPA and diagnostic disclosure at the memory clinic	D: qualitative P: 14 memory clinic patients and 13 family members S: three hospital-based clinics	M: focus groups O: experiences with NPA and diagnostic disclosure	The following themes were identified: uncertainty, early diagnostic paradox (both positive and negative experiences with NPA and diagnostic disclosure) and knowledge utilization (low information retention).
Holst et al., 2009 Research paper	Investigate patient's recollections of satisfaction with feedback after an NPA.	D: quantitative, descriptive P: 32 patients with ADHD or autism spectrum disorder S: 2 psychiatric outpatient clinics	M: survey O: satisfaction with feedback, self-perceived health, basic and earning self-esteem	Low levels of in-person feedback was related to low self-esteem. The more satisfied group had better physical and mental health, felt confirmed by the examiner, and had higher basic self-esteem.
Kirkwood et al., 2016 Research paper	Examine efficacy of a neuropsychological consultation as intervention for youth with persistent post-concussive symptoms following mild TBI	D: quantitative, non- randomized P: 80 children with MTBI and their parents S: outpatient pediatric concussion program	M: survey 1 week before and 1 and 3 months after NPA I: neuropsychological consultation with direct feedback O: post-concussive symptom rating	Significant decrease in post concussive symptoms 1 week and 3 months after in-person feedback in both parents and children. All received a summary of the report.
Kirkwood et al., 2017 Research paper	Examine parent satisfaction with neuropsychological consultation following MTBI in school-age children	D: quantitative, descriptive P: 71 parents of children with MTBI S: outpatient pediatric concussion program	M: survey O: satisfaction with NPA	Majority satisfied with NPA. A total of 93% found in-person feedback helpful and the majority thought feedback helped them understand problems (88%), strengths (86%), and ways to address problems (72%).
Lanca et al., 2019 Research paper	Examine self-reported cognitive and psychiatric symptoms, self-efficacy, motivation, and satisfaction following NPA with interventional feedback session	D: quantitative, descriptive P: 31 patients with ADHD or mood disorders S: neuropsychology outpatient clinic (community hospital)	M: survey O: satisfaction with NPA, psychiatric and cognitive symptoms, perceived memory ability, self-efficacy	One month after in-person feedback, reduction in psychiatric and cognitive symptoms was reported, as well as improved cognition and self-efficacy. Furthermore, high levels of satisfaction with NPA were reported.
Longley et al., 2012 Protocol	(1) investigate psychological benefit of NPA with feedback for patients with MS and their main caregivers following the feedback session and 2 months later (2) identify characteristics of patients and caregivers who will most benefit from assessment with feedback	P: patients with MS S: MS center	M: survey I: NPA + feedback, control group sham waiting list O: knowledge of cognitive profile, coping	Protocol for randomized controlled trial (study results to date yet unpublished; recruitment status: stopped early).
Lopez et al., 2008 Research paper	Outline a short intervention (three-session assessment and feedback module that translate NPA findings to daily life and eating symptoms) designed to identify and address information processing bias (poor set-shifting or strong detail focus) in patients with anorexia nervosa	D: qualitative (case study) P: 2 patients with anorexia nervosa S: outpatient clinic	N/A	In-person feedback led to adapted behavior in one patient and stopped rapid weight loss in the other patient.
Malla et al., 1997 Research paper	Determine whether detailed assessment of cognitive functions in people suffering from psychotic disorder can assist in their psychosocial rehabilitation while they are living in the community	D: qualitative (case study) P: 3 patients with schizophrenia S: community treatment program	N/A	In-person feedback in three patients led to improved outcomes by emphasizing strengths. In one patient it also led to improved feelings of self-esteem.
Martin & Schroeder, 2020 Research paper	Present survey results on how neuropsychologists approach feedback about invalid testing across various clinical settings.	D: quantitative, descriptive P: 209 clinical neuropsychologists S: various settings	M: survey O: base rate of invalidity, present three clinical case vignettes and inquire how they would give feedback	The majority (98%) of psychologists would include description of invalid findings and provide explanations (67%). There was little agreement on the approach in delivering feedback and what the goal was of the feedback session.
Meth et al., 2016 Research paper	Test a simple intervention (providing supplemental letter) in communicating neuropsychological feedback. Additionally, investigates the impact of including caregivers in the feedback session to explore whether differences exist in recall for recommendation between patients and family members	D: quantitative, randomized P: 79 patients with various diagnosis (most common psychiatric diagnosis, mild cognitive impairment, dementia) and 36 caregivers S: outpatients from neuropsychology lab (hospital)	M: telephone interview 7 weeks after NPA I: group with letter (n = 35) and group with no-letter (n = 44) O: retention and adherence to recommendations	Recall of recommendations was better in caregivers (not in patients) in the group with in-person and a supplemental letter compared to no letter. Overall level of recommendations remained low. No differences in adherences were found between the two groups.
Meth et al., 2019 Research paper	Identify types of recommendations that neuropsychologists provide to patients, and determine which specific recommendations are most and least consistently given to patients across and within different diagnostic populations	D: quantitative, descriptive P: 309 licensed clinical psychologists	M: survey O: type/frequency of recommendations	Most given recommendations across diagnoses were compensatory strategies, address cognitive deficits and to improve health. Psychologists were more likely to give recommendations that could be carried out without help from external sources.
Postal & Armstrong, 2013 Book	Reflect on how to communicate neuropsychological assessment results.	D: qualitative P: 82 psychologists	M: semi-structured interviews	Framework given on how to make in-person feedback stick (Table 3). Furthermore, concrete examples are given on how to give feedback on specific aspects based on the interviews with the psychologists.
Pritchard et al., 2014 Research paper	Preliminary evaluation of added value associated with NPA in the identification and treatment of ADHD in youth	D: quantitative, non-randomized P: 188 parents of children with ADHD S: neuropsychology outpatient clinic (hospital)	M: survey after NPA and after 5 months I: children who recently received NPA (NP+) and those who have not (NP-) O: parent rating of child symptoms, parent rating of child quality of life, satisfaction with NPA	Both patients with and without NPA reported significant improvements in the child’s behavioral and emotional symptoms. Feedback was seen as helpful and satisfaction with NPA was high.
Quillen et al., 2011 Research paper	To determine whether neuropsychological recommendations were implemented by families and whether the suggestions improved the survivor's quality of life (as perceived by parents) and school experience	D: quantitative, descriptive P: 20 parents of childhood history of cancer S: oncology center (hospital)	M: survey O: implementation of recommendations, improvement of parent perceived child quality of life	Most recommendations were academic or educational in nature and 48% were followed-up. All parents at least followed one recommendation, but adherence ranged between 16-100%. A total of 97% of the recommendations were seen as helpful in improving parent-perceived child quality of life.
Rosado et al., 2018 Research paper	Examine the impact of patient feedback regarding neuropsychological testing on patient outcomes	D: quantitative, non-randomized P: 84 patients with various diagnosis	M: survey at baseline and 6-8 weeks later semi-structured interview I: patients who attended feedback sessions (n =49) versus those who did not receive feedback (n=35) O: perceived stress, understanding of condition, coping	Patient with in-person feedback had increased quality of life, understanding of condition, ability to cope with condition compared to the group without feedback.
Ruppert & Attix, 2014 Book chapter	Brief summary of purposes and recommended practices for providing feedback on cognitive test results to patients and caregivers	P: patients with a cognitive impairment	N/A	Recommendations given for in-person feedback, such as clear communication, querying understanding throughout session, allowing patients/family to take notes, and provide written materials.
Stimmel et al., 2019 Research paper	Explore rates of adherence to neuropsychological recommendations, reasons for nonadherence, and the effect of oral (phone call) and written feedback over written feedback alone	D: quantitative, descriptive P: 55 patients with MS S: MS center (medical center)	M: semi-structured interview, retrospective record review O: adherence to treatment recommendations	Self-reported adherence to recommendations was 38%, but this varied per recommendation type (high adherence for pharmacological management and lower for psychotherapy or psychiatry referral/cognitive rehabilitation). Reasons for nonadherence were needing more information and wanting to speak with physician.
Tharinger & Pilgrim, 2012 Research paper	Evaluate whether receiving developmentally appropriate feedback in the form of individualized stories would affect how children and parents reported experiencing an NPA	D: quantitative, randomized P: 32 parents of children with primarily ADHD, CAPD or dysgraphia S: private practice	M: survey I: group with feedback following standard procedure (n = 17) and in experimental group with addition of child feedback through a fable (n =15) O: parent and child experience with NPA, satisfaction with NPA	Children who received in-person feedback with illustrative stories in a booklet experienced a greater sense of learning about themselves, more positive relationship with the assessor, and a sense that their parents learned more about them. Parents of these children reported a more positive relationship with their child and assessor, a greater sense of collaboration, and a higher level of satisfaction.
Turner et al., 2012 Research paper	Evaluate the feasibility of providing comprehensive neuropsychological evaluations and feedback via telemedicine to veterans receiving services from an urban veteran medical center	D: quantitative, non-randomized P: 15 veterans with cognitive or psychiatric disorders S: urban veteran medical center	M: informal questions during NPA I: group with in-person evaluation (n = 7), and group via telemedicine (n =8) O: satisfaction with NPA	Both groups were satisfied with receiving in-person feedback of feedback via telemedicine. NPA via telemedicine was deemed to be feasible and comparable to an in-person evaluation.
Westervelt et al., 2007 Research paper	Assess patient perceptions of neuropsychological evaluations. Furthermore, evaluated responses to neuropsychological recommendations.	D: quantitative, descriptive P: 129 patients with various diagnosis and 80 family members S: academic medical center neuropsychology program	M: survey (37% response rate) O: satisfaction with NPA	Patients were satisfied with NPA and receiving in-person feedback. Most were satisfied with the length of the feedback session and reported that it helped them understand problems, deal with problems, and reduce stress.

*NPA* neuropsychological assessment, *N/A* not applicable, *ADHD* attention deficit hyperactivity disorder, *MTBI* mild traumatic brain injury, *MS* multiple sclerosis

### Type and Quality of Evidence

The following 41 publications were identified: 26 research papers, seven opinion papers, two position papers, three book chapters, two books, and one research protocol. In terms of study designs, the research papers included four qualitative studies, three randomized trials, five non-randomized trials, thirteen descriptive studies, and one mixed-methods study. Only the overall quality of the research papers could be assessed (see Table 4 Appendix B). Overall, the quality criteria were met; in some studies, subitems were not met (e.g., due to insufficient detailed information about the completeness of the data). In two studies, there was also a high risk for a nonresponse bias due to low response rates.

### Characteristics of Neuropsychological Feedback

Most research papers used in-person feedback (Arffa & Knapp, 2008; Cheung et al., 2014; Connery et al., 2016; Fallows & Hilsabeck, 2013; Farmer & Brazeal, 1998; Foran et al., 2016; Holst et al., 2009; Kirkwood et al., 2016, 2017; Lanca et al., 2019; Lopez et al., 2008; Malla et al., 1997; Meth et al., 2016; Rosado et al., 2018; Tharinger & Pilgrim, 2012; Westervelt et al., 2007; Gruters et al., 2020; Martin & Schroeder, 2020), and most of the other publications recommended in-person feedback (Allen et al., 1986; Carone et al., 2010; Carone et al., 2013; Carone, 2017; Crosson, 2000; Gass & Brown, 1992; Gorske, 2007; Gorske & Smith, 2009; Green, 2000; Griffin & Christie, 2008; Postal & Armstrong, 2013; Ruppert & Attix, 2014). A minority gave feedback via phone (Stimmel et al., 2019), via telemedicine (Clement et al., 2001; Turner et al., 2012) or via a written report (Evans et al., 2019). Usually, feedback was given by the neuropsychologist a few weeks after the assessment. In four studies, this was given directly after testing (Meth et al., 2016; Kirkwood et al., 2016; Kirkwood et al., 2017; Connery et al., 2016).

The length and content of the feedback session was often not specified; when specified, it usually took approximately one hour and focused on cognitive strengths and weaknesses, the impacts of emotional functioning, the translation of results to daily life, and diagnostic issues and recommendations with compensatory strategies. A review of the included papers showed that neuropsychological feedback was provided for a wide spectrum of diagnoses (e.g., psychiatric, neurodevelopmental, brain injury, dementia, epilepsy, brain tumor). Most settings were neuropsychological outpatient clinics in a hospital. Whether neuropsychological feedback was always part of standard routine was not often specified. Two survey studies found that 26-44% of the patients received neuropsychological feedback and that the majority of all participants wanted more information (Bennett-Levy et al., 1994; Foran et al., 2016).

### Satisfaction with Neuropsychological Services

Approximately half of the research papers (k = 13), mostly survey studies, focused on experiences and satisfaction with the NPA. Overall, high levels of satisfaction with the NPA and feedback sessions were described for both patients and family members (Connery et al., 2016; Foran et al., 2016; Kirkwood et al., 2016; Kirkwood et al., 2017; Pritchard et al., 2014; Tharinger & Pilgrim, 2012; Turner et al., 2012; Westervelt et al., 2007). Patients were more satisfied with the NPA when they received feedback, and if they experienced this as useful (Bennett-Levy et al., 1994). In one qualitative study, it was found that both positive (relief or confirmation due to NPA outcome and diagnosis) and negative experiences (feeling distressed due to awareness of cognitive complaints) coexisted during an NPA and diagnostic disclosure at a memory clinic (Gruters et al., 2020). The highest utility ratings of NPA were related to understanding cognitive strengths, weaknesses, and the relation between results and everyday behavior (Arffa & Knapp, 2008). Sometimes feedback was a mere confirmation of suspicions, but this was also seen as helpful (Westervelt et al., 2007). Other predictors of NPA satisfaction included perceptions of professional competence and rating of neuropsychological recommendations (Farmer & Brazeal, 1998). Only in a minority of the studies were participants less satisfied with the feedback session (Bodin et al., 2007; Foran et al., 2016). In one study, participants felt that feedback did not provide as much help as they had expected (Bodin et al., 2007). In another study, participants criticized neuropsychological feedback, because the results were difficult to understand, expressing the need for additional feedback sessions (Foran et al., 2016). Holst et al. (2009) found that low levels of satisfaction were related to low levels of self-esteem. Participants who were more satisfied were able to develop a more positive relationship with the examiner (Holst et al., 2009).

### Recommendations Given in Neuropsychological Feedback

Both the research papers, opinion/position papers, books, and book chapters showed that the recommendations given most often by psychologists across diagnoses were focused on compensatory strategies, cognitive deficits, and health improvements. A survey study showed that most psychologists gave feedback and explanations about invalid test results (Martin & Schroeder, 2020). Quantitative studies showed that differences in recommendations were identified between diagnoses (e.g., more focused on support/independence or driving in dementia and on rehabilitation referrals in patients with traumatic brain injury) (Meth et al., 2019; Quillen et al., 2011). On average, six primary and eleven secondary recommendations were given (Arffa & Knapp, 2008). The majority of participants were positive and experienced recommendations as helpful (Quillen et al., 2011; Cheung et al., 2014). However, the overall adherence to recommendations was found to be approximately 40% in four different quantitative studies (Quillen et al., 2011; Cheung et al., 2014; Stimmel et al., 2019; Westervelt et al., 2007). Identified barriers were unwillingness to adapt to the recommendations of the family member, patient misunderstandings, a need for more information, disagreement with recommendations, a desire to speak with a physician regarding the recommendations, and level of difficulty obtaining recommended services (Cheung et al., 2014; Stimmel et al., 2019; Westervelt et al., 2007). Higher adherence rates were found for pharmacological management and recommendations related to patient safety (Westervelt et al., 2007; Stimmel et al., 2019).

### Information Provision During Neuropsychological Feedback

One quantitative study and one mixed-methods study showed that neuropsychological feedback was not always remembered or understood (Bennett-Levy et al., 1994; Foran et al., 2016). Two randomized controlled trials showed that offering supplemental written information improved the free recall of recommendations (Meth et al., 2016; Fallows & Hilsabeck, 2013), especially in family members (Meth et al., 2016). However, the recall of diagnostic information did not differ between the groups with and without supplemental written information (Fallows & Hilsabeck, 2013; Meth et al., 2016). One survey study found that only one-third of the participants received a written report (Bennett-Levy et al., 1994), while in three studies (two survey studies, one non-randomized trial), it was found that the patients experienced the written report as helpful (Farmer & Brazeal, 1998; Bennett-Levy et al., 1994; Pritchard et al., 2014). A qualitative study and one opinion paper identified the following barriers in report writing: difficult to understand or unhelpful information, language proficiency, level and quality of education. It is important to consider ethnicity, country, native tongue, literacy, educational attainment and culture of origin, as these factors may contribute to barriers in understanding the feedback or terminology used, as well as treatment adherence. (Evans et al., 2019; Griffin & Christie, 2008). Some recommendations offered for report writing by these authors were to use as little information as possible, use in-text formatting, and organize headings by audience, diagnosis, and recommendations. Further advice was to be aware of cultural and linguistic differences, transparency, and translation of scores to daily life (Evans et al., 2019).

In one randomized controlled trial, a group of children and parents who received feedback with illustrative individualized stories reported that children experienced a greater sense of learning and collaboration, and a more positive relationship with the examiner compared to children and parents that received feedback without these stories. Parents experienced a greater sense of collaboration and a more positive relationship with their child and the examiner; they also reported higher satisfaction with the NPA (Tharinger & Pilgrim, 2012). No other studies focused on using visual aids in neuropsychological feedback; however, in one case study, one book, and two opinion papers, visual aids were recommended (Lopez et al., 2008; Postal & Armstrong, 2013; Carone et al., 2010; Gass & Brown, 1992). The use of props (e.g., brain model) or several shorter feedback sessions was recommended in two publications (Postal & Armstrong, 2013; Carone et al., 2013; Gass & Brown, 1992).

Postal and Armstrong (2013) described six principles of improving retention of neuropsychological feedback in their book: (1) simplicity (core message), (2) unexpectedness (novel information is better remembered), (3) concreteness (translation to daily life), (4) credibility (trusted source), (5) emotions (enhancing effects of emotions on memory), and (6) stories (transform passive listeners to active imaginers). They also advocate using motivational interviewing.

### Neuropsychological Feedback and Patient Outcomes

Eight research papers explored the impact of neuropsychological feedback on patient outcomes. Patients with a mood disorder or ADHD reported reductions in psychiatric and cognitive symptoms and improvements in self-efficacy for general- and evaluation-specific goals one month after an NPA with feedback (Lanca et al., 2019). Two case studies of patients with anorexia nervosa or schizophrenia reported that feedback gave patients insight into their cognitive functioning so that they could deal with their disease in a different way in their daily life (Lopez et al., 2008; Malla et al., 1997). Patients who attended neuropsychological feedback sessions had a greater improvement in quality of life and an increased understanding of and ability to cope with their condition compared to those who did not attend these sessions (Rosado et al., 2018). Two studies found a decrease in self-reported post-concussive symptoms in both parents and children (Connery et al., 2016; Kirkwood et al., 2016). Greater initiation of parent behavior management and special education services and medication management were reported when parents of children with ADHD received neuropsychological feedback (Pritchard et al., 2014). After receiving feedback, the concerns of family members of patients with a stroke were related to safety, what the future will bring, knowing what to do when the patient is unable to perform a task, and dealing with the emotional changes of the patient (Belciug, 2006).

### Feedback Frameworks and Clinical Recommendations

Table 2 shows an overview of the different feedback frameworks offered by six authors from one non-randomized study, three opinion papers, and two books. In all frameworks, some overlap can be identified: an explanation of the nature of NPA and rationale of feedback sessions, an explanation of strengths and weaknesses, and the provision of recommendations and compensatory strategies. Furthermore, in these feedback models, a collaborative approach using plain and understandable language without jargon is advocated.

**Table 2:** Frameworks for providing neuropsychological feedback in different settings

Author	Patient group	Phases	Recommendations
Carone et al., 2013; Carone et al., 2010	MTBI patients with invalid effort.	1) Build rapport and obtain informed consent 2) Preliminary discussion 3) Feedback session	Avoid accusatory and emotionally laden terms Ask their input first Explain strengths and weaknesses Explain incredible efforts as good/bad news
Carone, 2017	Patients with high level of cognitive complaints, but normal test performance.	1) Build rapport and obtain informed consent 2) Let patients self-rate performance 3) Feedback session	Show patient a table with the objective and self-reported performance on cognitive tests
Connery et al., 2016	Children with invalid effort.	1) Opening statement invalid effort 2) Emphasize importance of performance validity testing 3) Give recommendations	Give feedback to parents first Give a brief explanation to children
Gass & Brown, 1992	Patients with brain injuries and their family members	1) Review purpose of testing 2) Define the tests 3) Explain results per cognitive domain 4) Describe strengths and weaknesses 5) Address diagnostic/prognostic issues 6) Give recommendations	Use plain and understandable language Use behavioral/concrete examples for explaining tests Explain normative comparison Ask for feedback after each domain
Gorske & Smith, 2009	Clinical setting	1) Set agenda and introduce feedback 2) Develop 2-3 life implication questions 3) Determine personal skill profile 4) Describe strengths and weaknesses 5) Summarize relationship between results, life areas and patient questions	Give in-person feedback Use Motivational Interviewing Principles ‘Elicit-Provide-Elicit’ Ask about central cognitive complaint Explain normative comparison Use graphic illustrations Provide copy of feedback report
Postal & Armstrong, 2013	Clinical setting	1) Reorient patient and family 2) Gather more information 3) Be flexible 4) Lead with the core message 5) Describe strengths and weaknesses 6) Give recommendations	Explain nature of session Review goal of patient and family Remind the patient of the collaborative process Explain normative comparison Use concrete metaphors for cognitive domains Avoid the use of jargon

## Discussion

This scoping review identified 41 publications on neuropsychological feedback to patients and family members in diverse settings. Several themes related to neuropsychological feedback could be identified: characteristics of neuropsychological feedback, satisfaction with neuropsychological services, patient outcomes, recommendations given in neuropsychological feedback, information provision during neuropsychological feedback, feedback frameworks and clinical recommendations. A critical evaluation of the methodological quality of the included research papers showed that most met the criteria, but not all intervention studies included a control group, not all were randomized, and not all described the sample or outcome data in detail. Most prominent was the risk of response bias in the cross-sectional survey studies as a result of low response rates.

The majority of publications recommended or used in-person feedback. Approximately thirty years ago, Pope (1992) stated that feedback was the most neglected part of psychologists’ assessments. In our review, one survey study and one mixed-methods study evaluating clinical practice showed that neuropsychological feedback was not given to all patients (Bennett-Levy et al., 1994; Foran et al., 2016). However, one of these studies was carried out over 25 years ago. When reviewing broader psychological assessment practices (e.g., also considering personality assessment), two survey studies showed that the majority of psychologists gave in-person feedback, although this was not always a routine part of their assessment (Smith et al., 2007; Curry & Hanson, 2010). Based on these results, it seems that psychologists currently give feedback in psychological diagnostic procedure more often than 25 years ago. However, it is still not a clinical routine, and the content of neuropsychological feedback depends on the clinical context. Nonetheless, the potential clinical benefits of psychological feedback have been demonstrated, such as improved self-esteem, hopefulness, and reduced symptoms (Ackerman et al., 2000; Allen et al., 2003; Finn & Tonsager, 1997). Giving feedback also led to a better therapeutic alliance in psychotherapy (Ackerman et al., 2000; Hilsenroth et al., 2004). Furthermore, a meta-analysis showed that only when psychological assessment procedures were combined with personalized and collaborative feedback did clinically meaningful effects on treatment emerge (Poston & Hanson, 2010) In another study it was shown that psychological test validity was indistinguishable from medical tests validity, and that assessment feedback was related to increased patient well-being (Meyer et al., 2001; Finn & Tonsager, 1992; Newman & Greenway, 1997). Although research is lacking on the therapeutic value of collaborative neuropsychological feedback, it is highly likely that it has similar effects as in other psychological assessment fields. However, when looking at multidisciplinary practices, the diagnostic disclosure (based on all diagnostic assessments) is often given only by the medical specialist. Offering an additional consultation with a neuropsychologist to discuss NPA findings in multidisciplinary practices could be helpful in patients and offers the opportunity to improve information retention and answer remaining questions. Furthermore, it offers the opportunity to provide support and guidance due to the emotional impact of the diagnosis.

Consumer experience and satisfaction with NPA were the most reported outcomes in the current scoping review. Generally, positive experiences and high satisfaction with neuropsychological procedures were reported. Gaining more insight into consumer experiences and satisfaction is relevant, as it may lead to improvement of the quality of service and patient outcomes, such as reduced patient anxiety due to good communication (van Osch et al., 2017). However, the validity of satisfaction in treatment outcomes has been criticized as a result of not taking psychological factors, communication, and patient expectations into account (Verbeek, 2004; Hudak et al., 2003). In terms of an NPA, patient satisfaction was related to receiving feedback that was evaluated as valuable (Bennett-Levy et al., 1994). This is in line with the findings of Smith et al. (2007), who showed that patients appreciated that psychological feedback helped understand their problems, which could result in positive changes. However, not understanding or remembering neuropsychological feedback might negatively influence satisfaction. Therefore, Brenner (2003) has developed a framework that psychologists can use to increase the beneficial value of and satisfaction with psychological assessments: elimination of jargon, focus on referral questions, individualized assessment reports, emphasis on patient strengths, and including concrete recommendations.

The current review also showed that information retention of neuropsychological feedback was low. A few quantitative studies reported low percentages of information retention, especially recommendations were difficult to remember (Meth et al., 2016). This is in line with earlier studies that showed that the retention of medical information was generally low (Kessels, 2003). This is alarming, as adherence to treatment recommendations is related to understanding, recall, and satisfaction with the consultation (Ley, 1979). Understanding health information, also defined as health literacy, is important because low health literacy is the strongest predictor of poor health outcomes, such as more hospital admissions, higher mortality rates, and inadequate medication adherence (Berkman et al., 2011), as well as poor disease or care management (Graham & Brookey, 2008). Furthermore, patients with a low health literacy are less likely to take preventive measures or adopt a healthy lifestyle (Graham & Brookey, 2008). Remembering and understanding neuropsychological feedback may be even more difficult in patients who have a cognitive impairment. Retention might also be hampered by emotional arousal or valence related to receiving (*bad news)* or not receiving (*good news)* a diagnosis (Kensinger, 2009). On the one hand, receiving a ‘bad news’ diagnosis has profound emotional effect on both the patient and family members, such as feelings of stress and anxiety. It is very likely that this leads to lower recall of information. On the other hand, feeling relieved if the feared diagnosis is not confirmed might also negatively influence information retention. A few studies have explored the mode of information as a suitable target for communication aids. The current scoping review showed that offering supplemental written information might be helpful, but no studies have been conducted on whether visual aids could improve information retention in neuropsychological feedback. Some promising findings were seen in other fields, such as improved recall when using three-dimensional MRI models of the tumor and brain compared to two-dimensional models (Sezer et al., 2020). Furthermore, a systematic review showed better recall in multiple studies when visual aids were used (Watson & McKinstry, 2009). It is essential that neuropsychological feedback can be remembered so that it can lead to improved treatment outcomes. This review also showed that Motivational Interviewing principles might improve information retention as suggested by Postal and Armstrong (2013). In addition, motivational interviewing may also improve treatment adherence in rehabilitation, psychotherapy, and medication (Palacio et al., 2016; Tolchin et al., 2019; Suarez, 2011). Furthermore, the use of these techniques has been shown to reduce addictive behaviors (e.g., binge drinking, alcohol consumption) (Frost et al., 2018). Other applications of motivational interviewing have also been described, such as dealing with patient resistance (Gorske, 2007). By using these principles patients might understand the information and be more likely to change their behavior.

A strength of the current study is its use of the scoping review methodology to gain insight into a broad spectrum of literature on providing neuropsychological feedback. It synthesizes evidence on an emerging topic while using a systematic process that is both replicable, transparent and rigorous. Furthermore, the review has been carried out by experts in the field of clinical neuropsychology with the involvement of an expert librarian. Most scoping reviews do not include a critical appraisal of the quality of the included studies. However, we have included this to not only describe the scope of the research questions but also to give an impression of the validity.

Some limitations must be mentioned as well. One limitation is that reviews are time consuming, and new publications might have emerged since the search. However, we have recently updated the search, which resulted in the inclusion of two full papers. A second limitation is that although we have tried to discuss the most important findings, due to the limited space, some details might have not been described. Furthermore, the variation between the document types and range of data collection, methodology and analysis techniques made it sometimes difficult to present the results in a compact and integrated way.

Finally, future research should include more studies adopting a randomized controlled trial to gain more insight into the benefits of neuropsychological feedback. It would also be of interest to evaluate neuropsychological feedback in specific settings with a multidisciplinary nature, such as memory clinics. Furthermore, more attention is needed to train psychologists in providing neuropsychological feedback. It would be helpful if more attention could be given about this topic during graduate and postgraduate education courses of psychologists. The clinical recommendations offered in this paper could be used. Finally, more research is also needed focusing on how neuropsychological feedback could be best communicated to optimize information retention and treatment adherence. More research is needed to identify predictors of improved information retention (e.g., having multiple sessions, involving a family member, use of motivational interviewing principles). Furthermore, more studies are needed on how to best offer supplemental verbal or visual information to improve comprehension and retention of neuropsychological feedback.

## Conclusion

Overall, the current scoping review shows that neuropsychological feedback is a vital and therapeutic component that may improve patients’ satisfaction with neuropsychological services. However, the content, frequency and type of feedback vary greatly across professionals. Furthermore, it should be stressed that information retention and adherence to recommendations is often poor, despite the provision of neuropsychological feedback. It is important that clinicians are aware of facilitators and barriers in offering neuropsychological feedback. In Table 3 we provide an overview of clinical recommendations is presented that can be used to improve retention, adherence, and the clinician-patient relationship through motivational interviewing principles. Although using communication aids seems promising, more research is warranted.

**Table 3:** Clinical recommendations

Improve retention	• Give in-person feedback, plan multiple sessions if needed • Give balanced feedback (focus on strength and weaknesses) • Explain the normative comparison • Use concrete metaphors to illustrate cognitive domains • Limit feedback to essential points and repeat these • Involve family members • Let patients and family members take notes • Provide written or visual materials using understandable language. • Evaluate level of understanding and level of emotional acceptance multiple times.
Improve adherence	• Ascertain the retention of the feedback provided • Evaluate whether the patient is willing to adapt to recommendations and explore barriers they might experience (e.g., access to care) • Communicate with the referrer to attenuate whether feedback and recommendations are appropriate • Provide patient and family with contact details if questions arise
Motivational Interviewing (MI)	MI principles can be used to improve retention, adherence and a positive relationship with the clinician. The core principles: • Show empathy (e.g., reflective listening principles such as listening rather than telling) • Make the patients see the discrepancy between their behavior and their goals (e.g., by making them aware of the consequences) • Avoid argument (e.g., by shifting the attention of focus on another topic) • Do not oppose to resistance, but adjust to the patient • Stimulate self-efficacy

## Appendix A Search Strategy

### Pubmed

('neuropsychological assessment'[Title/Abstract] OR 'neuropsychological evaluation'[Title/Abstract] OR 'neuropsychology'[Title/Abstract] OR 'neuropsychologist'[Title/Abstract] OR 'neuropsychological'[Title/Abstract] OR neuropsychological test*[Title/Abstract] OR neuropsychological result*[Title/Abstract] OR "Neuropsychology/instrumentation"[Mesh] OR "Neuropsychology/methods"[Mesh] OR "Neuropsychology/organization and administration"[Mesh] OR "Neuropsychology/standards"[Mesh] OR "Neuropsychology/trends"[Mesh] OR "Neuropsychological Tests/instrumentation"[Mesh] OR "Neuropsychological Tests/methods"[Mesh] OR "Neuropsychological Tests/standards"[Mesh]) AND (feedback[Title/Abstract] OR recommendations[Title/Abstract] OR communicat*[Title/Abstract] OR "Feedback/instrumentation"[Mesh] OR "Feedback/methods"[Mesh] OR "Feedback, Psychological/instrumentation"[Mesh] OR "Feedback, Psychological/methods"[Mesh] OR "Feedback, Psychological/organization and administration"[Mesh] OR "Feedback, Psychological/standards"[Mesh] OR "Feedback, Psychological/trends"[Mesh] OR "Communication/diagnosis"[Mesh] OR "Communication/instrumentation"[Mesh] OR "Communication/methods"[Mesh] OR "Communication/organization and administration"[Mesh] OR "Communication/psychology"[Mesh] OR "Communication/standards"[Mesh] OR "Communication/trends"[Mesh]) AND (patient OR family members OR carers)

### PsycInfo/CINAHL

(AB 'neuropsychological assessment' OR AB ‘neuropsychological evaluation’ OR AB ‘neuropsychology’ OR AB ‘neuropsychologist’ OR ‘AB ‘neuropsychological’ OR AB ‘neuropsychological test*’ OR AB ‘neuropsychological result*’ OR DE ‘neuropsychology’ OR DE ‘neuropsychological assessment’) AND

(AB ‘feedback’ OR AB ‘recommendations’ OR AB ‘communicat*’ OR DE ‘feedback’ OR DE ‘communication’) AND (‘patient’ OR ‘family members’ or ‘carers’)

### Embase

(*neuropsychology/ or *neuropsychological test/ or (neuropsychological test or neuropsychological evaluation or neuropsychology or neuropsychologist or neuropsychological or neuropsychological test* or neuropsychological result*).ti,ab.) and (*psychological feedback/ or (feedback or recommendations or communicat*).ti,ab.) and (patient or family members or carers).mp.

### Web of Science

TI=(neuropsychological assessment OR neuropsychological evaluation OR neuropsychology OR neuropsychologist OR neuropsychological OR neuropsychological test* OR neuropsychological result*) AND TS=(feedback OR recommendations OR communicat*) AND ALL=(patient OR family members OR carers)

## Appendix B Quality Assessment

**Table 4:** Quality assessment (based on the Mixed Model Appraisal Tool) of included research papers (k = 26)

Author(s) (year)	Study type	Criteria	Criteria met by studies		
			Yes	No	Not enough information
Cheung et al. (2014)	Qualitative	1.1 Relevant qualitative approach	X		
		1.2 Adequate qualitative data collection	X		
		1.3 Findings adequately derived from data	X		
		1.4 Adequate interpretation of results	X		
		1.5 Coherence between data	X		
Gruters et al. (2020)	Qualitative	1.1 Relevant qualitative approach	X		
		1.2 Adequate qualitative data collection	X		
		1.3 Findings adequately derived from data	X		
		1.4 Adequate interpretation of results	X		
		1.5 Coherence between data	X		
Lopez et al. (2008)	Qualitative	1.1 Relevant qualitative approach	X		
		1.2 Adequate qualitative data collection	X		
		1.3 Findings adequately derived from data	X		
		1.4 Adequate interpretation of results	X		
		1.5 Coherence between data	X		
Malla et al. (1997)	Qualitative	1.1 Relevant qualitative approach	X		
		1.2 Adequate qualitative data collection	X		
		1.3 Findings adequately derived from data	X		
		1.4 Adequate interpretation of results	X		
		1.5 Coherence between data	X		
Fallows & Hilsabeck (2013)	Quantitative randomized	2.1 Appropriate randomization			X
		2.2 Comparable groups at baseline	X		
		2.3 Complete outcome data	X		
		2.4 Outcome assessors blind		X	
		2.5 Adherence to intervention	X		
Meth et al. (2016)	Quantitative randomized	2.1 Appropriate randomization	X		
		2.2 Comparable groups at baseline	X		
		2.3 Complete outcome data	X		
		2.4 Outcome assessors blind	X		
		2.5 Adherence to intervention	X		
Tharinger and Pilgrim (2012)	Quantitative randomized	2.1 Appropriate randomization	X		
		2.2 Comparable groups at baseline	X		
		2.3 Complete outcome data	X		
		2.4 Outcome assessors blind		X	
		2.5 Adherence to intervention	X		
Connery et al. (2016)	Quantitative non-randomized	3.1 Representative sample	X		
		3.2 Appropriate measures	X		
		3.3 Complete outcome data	X		
		3.4 Confounders accounted for	X		
		3.5 Intervention administered as intended	X		
Rosado et al. (2018)	Quantitative non-randomized	3.1 Representative sample	X		
		3.2 Appropriate measures	X		
		3.3 Complete outcome data	X		
		3.4 Confounders accounted for			X
		3.5 Intervention administered as intended	X		
Turner et al. (2012)	Quantitative non-randomized	3.1 Representative sample	X		
		3.2 Appropriate measures		X	
		3.3 Complete outcome data			X
		3.4 Confounders accounted for		X	
		3.5 Intervention administered as intended	X		
Kirkwood et al. (2017)	Quantitative non-randomized	3.1 Representative sample	X		
		3.2 Appropriate measures	X		
		3.3 Complete outcome data	X		
		3.4 Confounders accounted for	X		
		3.5 Intervention administered as intended	X		
Pritchard et al. (2014)	Quantitative non-randomized	3.1 Representative sample	X		
		3.2 Appropriate measures	X		
		3.3 Complete outcome data	X		
		3.4 Confounders accounted for	X		
		3.5 Intervention administered as intended	X		
Arffa and Knapp (2008)	Quantitative descriptive	4.1 Sampling strategy relevant	X		
		4.2 Representative sample	X		
		4.3 Appropriate measurements	X		
		4.4 Low risk of nonresponse bias	X		
		4.5 Appropriate statistical analysis	X		
Belciug (2006)	Quantitative descriptive	4.1 Sampling strategy relevant	X		
		4.2 Representative sample			X
		4.3 Appropriate measurements	X		
		4.4 Low risk of nonresponse bias	X		
		4.5 Appropriate statistical analysis	X		
Bennet-Levy et al. (1994)	Quantitative descriptive	4.1 Sampling strategy relevant	X		
		4.2 Representative sample	X		
		4.3 Appropriate measurements	X		
		4.4 Low risk of nonresponse bias	X		
		4.5 Appropriate statistical analysis	X		
Farmer and Brazeal (1998)	Quantitative descriptive	4.1 Sampling strategy relevant	X		
		4.2 Representative sample	X		
		4.3 Appropriate measurements	X		
		4.4 Low risk of nonresponse bias		X	
		4.5 Appropriate statistical analysis	X		
Holst et al. (2009)	Quantitative descriptive	4.1 Sampling strategy relevant	X		
		4.2 Representative sample	X		
		4.3 Appropriate measurements	X		
		4.4 Low risk of nonresponse bias	X		
		4.5 Appropriate statistical analysis	X		
Lanca et al. (2019)	Quantitative descriptive	4.1 Sampling strategy relevant	X		
		4.2 Representative sample	X		
		4.3 Appropriate measurements	X		
		4.4 Low risk of nonresponse bias	X		
		4.5 Appropriate statistical analysis	X		
Martin and Schroeder (2020)	Quantitative descriptive	4.1 Sampling strategy relevant	X		
		4.2 Representative sample	X		
		4.3 Appropriate measurements	X		
		4.4 Low risk of nonresponse bias	X		
		4.5 Appropriate statistical analysis	X		
Meth et al. (2019)	Quantitative descriptive	4.1 Sampling strategy relevant	X		
		4.2 Representative sample	X		
		4.3 Appropriate measurements	X		
		4.4 Low risk of nonresponse bias	X		X
		4.5 Appropriate statistical analysis	X		
Quillen et al. (2011)	Quantitative descriptive	4.1 Sampling strategy relevant	X		
		4.2 Representative sample	X		
		4.3 Appropriate measurements			X
		4.4 Low risk of nonresponse bias	X		
		4.5 Appropriate statistical analysis			X
Stimmel et al. (2019)	Quantitative descriptive	4.1 Sampling strategy relevant	X		
		4.2 Representative sample	X		
		4.3 Appropriate measurements	X		
		4.4 Low risk of nonresponse bias	X		
		4.5 Appropriate statistical analysis	X		
Westervelt et al. (2007)	Quantitative descriptive	4.1 Sampling strategy relevant	X		
		4.2 Representative sample	X		
		4.3 Appropriate measurements	X		
		4.4 Low risk of nonresponse bias	X		
		4.5 Appropriate statistical analysis	X		
Bodin et al. (2007)	Quantitative descriptive	4.1 Sampling strategy relevant	X		
		4.2 Representative sample	X		
		4.3 Appropriate measurements	X		
		4.4 Low risk of nonresponse bias		X	
		4.5 Appropriate statistical analysis	X		
Kirkwood et al. (2017)	Quantitative descriptive	4.1 Sampling strategy relevant	X		
		4.2 Representative sample	X		
		4.3 Appropriate measurements	X		
		4.4 Low risk of nonresponse bias	X		
		4.5 Appropriate statistical analysis	X		
Foran et al. (2016)	Mixed-methods	5.1 Adequate rationale for mixed methods	X		
		5.2 Components adequately integrated	X		
		5.3 Components adequately interpreted	X		
		5.4 Differences addressed	X		
		5.5 Components adhere quality criteria	X		
